# Blockchain System in the Higher Education

**DOI:** 10.3390/ejihpe11010021

**Published:** 2021-03-16

**Authors:** Ricardo Raimundo, Albérico Rosário

**Affiliations:** 1 Higher Institute of Education and Sciences, 1750-142 Lisbon, Portugal; ricardo.raimundo@iseclisboa.pt; 2 Research Unit on Governance, Competitiveness and Public Policies (GOVCOPP), University of Aveiro, 3810-193 Aveiro, Portugal

**Keywords:** blockchain, education, higher education

## Abstract

Blockchain has emerged as an important concept at the interface of ICT and higher education. It is a system in which a record of transactions is maintained across several computers that are linked in a peer-to-peer network. Hence, it allows the creation of a decentralized environment, where data are not under the control of any third-party organization. This study presents a Systematic Bibliometric Literature Review (LRSB in further text) of research on blockchain applications in the higher education field. The review integrated 37 articles presenting up-to-date knowledge on current implications pertaining to the use of blockchain technology for improving higher education processes. The LRSB findings indicate that blockchain is being used to build up new interventions to improve the prevailing ways of sharing, delivering and securing knowledge data and personal student records. The application of blockchain technology is carrying on a conceptual progress in the higher education sector where it has added substantial value by ameliorated efficiency, effectiveness, privacy control, technological improvement and security of data management mechanisms. Challenges posed by current literature and further research directions are suggested.

## 1. Introduction

Blockchain was originally introduced as a system to control Bitcoin [1] but has now evolved to the point of being considered an introductory technology for various decentralized applications [2]. It is being publicized as a helpful technology for organizing sensitive data, especially within the sectors of higher education, healthcare, supply chain and the Internet of Things (IoT) [3]. Higher education may be understood as a system that includes, among others, two major stakeholders, Higher Education Institutions (HEIs in further text) and students [4]. 

In this study, we consider the contribution of blockchain in higher education regarding the use of IT infrastructure and computing solutions to monitor the multiple systems within a university, in an effort to promote, maintain or restore the educational system [5]. In the field of higher education, privacy and security breaches are purportedly increasing every year, especially with regard to academic diplomas and degrees. Blockchain technology has a role in ensuring their authenticity and keeping accurate records [6]. The increasing digitization of higher education has further led to the acknowledgment of concerns related to secure storage, while blockchain technology allows decentralized open data, absence of frauds, safe storage of information, and reduction in transaction expenses related to academic data control [7]. Blockchain has been suggested as a way to solve critical challenges faced by higher education, such as recordkeeping of diplomas and by a student-centric approach [8].

Nevertheless, prior research has made partial attempts to summarize existing knowledge through systematic literature reviews (SLRs) [5,7,9,10,11,12]. For example, some discuss the application of the latest key technologies for the development of smart campuses and universities [5]; the advantages of this technology as decentralized open data for safe storage of information in specific case studies (e.g., MIT) [7], the use of these technologies for assisting students in their acquisition of technical knowledge and development in Engineering [9] and the creation of distributed applications involving multiple actors without the control of a central authority [11].

Despite the fact that these SLRs have built the existing knowledge on the topic, their focus has mostly been on underscoring areas of Blockchain application [5,9]. Nonetheless, because of the extent and variety of existing research on blockchain, researchers would benefit from a focused discussion on the areas of improvement from its adoption [12]. Review-based studies can assist in achieving this goal by synthesizing extant knowledge and explaining key issues that need substantial focus [10]. In this way, the study is driven by two research questions: what is the research building for Blockchain technology in the higher education sector to date? Additionally, what are the current challenges and future research paths in the theme?

We address these research goals by carrying out a Systematic Bibliometric Literature Review (LRSB) on the use of blockchain in higher education [5]. LRSBs can offer an important synthesis of current knowledge in a research topic and unveil existing knowledge gaps and, ensuing directions for future research [9,12]. This study is built on the current literature on blockchain in higher education by adding to prior SLRs in two ways. First, it provides a bibliographically organized categorization of prior studies with regard to their application areas. Second, with an eye on the findings, we point out potential themes that demand more attention and major challenges and research paths to move forward the existing knowledge. This contribution is made by dividing this study into two key discussion sections: (a) the research building for Blockchain technology in the higher education sector to date; and (b) the current challenges and future research paths in the theme. 

The remainder of the article is structured as follows. Section 2 offers an overview of blockchain technology. Section 3 explains the methodology adopted for the current LRSB. The findings are provided in Section 4, followed by its discussion. Finally, Section 5 discusses concluding remarks, limitations and future research directions.

## 2. Blockchain Technology

Blockchain is perceived as a revolutionary technology offering considerable impact on a vast magnitude of sectors [3] which enables the creation of decentralized applications programmed to run on network and records sets of data that can be shared securely without third-party mediation [13]. In blockchain applications data are stored by encrypted group signature to, along with shared algorithms, solve the problem of anonymous abuse [14]. These features for data storage and validation are the key issue for the effective use of blockchain in higher education [15], in which a huge amount of data in terms of diplomas and degree certificates is exchanged among institutions [16]. 

As already mentioned, the evolution of blockchain technology started with cryptocurrency and has evolved towards the application of smart contracts in areas such as healthcare, supply chain, the Internet of Things (IoT), finance, voting systems, property and real-estate management, e-government, higher education, among others [3]. The Blockchain emphasis on variety in terms of applications may be due to its capacity to build a trusted [17] and decentralized contract environment [18]. 

The higher education sector is therefore a potential user for blockchain technology in terms of smart contracts, [19] due to its capacity in allowing stakeholders to validate learning records [20] and identity management, for instance [21]. This may allow institutions to decide with which other HEIs to share data, therefore avoiding that trustworthy qualifications (diplomas or certificates) may be counterfeited or falsified [22]. Additionally, its distributed ledger and lack of need for a trusted third party can improve smart contract-based protocols that automatically enforce a contract in students throughout multiple levels of administration, which constitutes a major advantage of blockchain for the higher education field [17]. Last but not least, this technology can ease processes while mitigating the probability of error [23].

Nevertheless, there seems to exist to some extent a fairly strong resistance to adoption of this technology, either because of some degree of doubt on its capacity to overcome administrative barriers through a more transparent and technologically advanced form of higher education systems [18], or due to some skepticism in terms of its capacity to unbundle higher education processes through a more effective student experience and enhanced faculty roles [24]. Finally, from the employers’ perspective, there is a lack of trust due to inaccuracies when students describe skills and qualifications in their resumes [20]. Hence, there is a strong need for more knowledge of blockchain and its complexity in order to bring light on the key advantages of blockchain both for students and HEIs, who often have inappropriate education and coordinating systems for preparing professional staff working, while using classic methods of learning and education techniques with no real connection with technology for data management [25]. 

Current research is focused on assisting the technological evolution of blockchain by dealing with these challenges.

## 3. Materials and Methods 

We carried out a LRSB of the “Blockchain system in Higher Education” [26,27,28]. As opposed to the alternatives of traditional literature revision and systematic revision, this research concept is based on a more careful perspective of the recognition and synthesis of information [26,27,28,29], improving: (i) the validity of the review, providing a set of steps that can be followed if the study is replicated; (ii) accuracy, providing and demonstrating arguments strictly related to research issues; and (iii) the generalizability of the results, allowing the synthesis and analysis of accumulated knowledge. Thus, LRSB is a “guiding instrument” that allows guiding the review according to the objectives.

The study is carried out as follows: (i) definition of the research question; (ii) location of studies; (iii) selection and evaluation of studies; (iv) analysis and synthesis; (v) presentation of results; lastly (vi) discussion and conclusion of results [26,27,28].

The methodology presented ensures that the review is comprehensive, auditable, and replicable and responds to research questions [26,27,28,29].

The review was carried out in December 2020, with a bibliographic search in the SCOPUS database of scientific articles published by 2020. The research was carried out in three phases: (i) using the keyword “blockchain”, 16,139 documents were obtained; (ii) adding the keyword “education”, a set of 473 documents were obtained; and finally (iii) keywords: “blockchain”, “education” and “higher education”, a set of 37 studies was obtained with a publication already confirmed for 2021, as listed below (Table 1).

## 4. Literature Analysis: Themes and Trends

**Figure 1:**
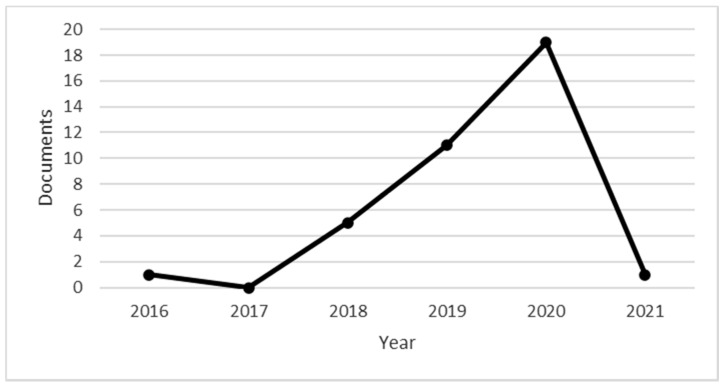
Documents by year. Source: own elaboration.

Peer-reviewed articles on the subject were selected until 2020. In the period under review, 2020 was the year with the highest number of peer-reviewed articles on the subject, with 19 publications, with one publication already confirmed for 2021. Figure 1 reviews peer-reviewed publications published until 2020.

The publications were sorted out as follows: ACM International Conference Proceeding Series 4 publications, with two publications (Communications in Computer and Information Science; Iberian Conference on Information Systems and Technologies Cisti; International Journal on Interactive Design and Manufacturing) with one publication (2018 World Engineering Education Forum Global Engineering Deans Council Weef Gedc 2018; 2019 42nd International Convention on Information and Communication Technology Electronics and Microelectronics Mipro 2019 Proceedings; 2019 4th Mec International Conference on Big Data and Smart City Icbdsc 2019; 2020 2nd Conference on Blockchain Research and Applications for Innovative Networks and Services Brains 2020; 2020 43rd International Convention on Information Communication and Electronic Technology Mipro 2020 Proceedings; Advances in Intelligent Systems and Computing; Applied Sciences Switzerland; Csedu 2020 Proceedings of the 12th International Conference on Computer Supported Education; European Journal of Contemporary Education; IEEE Access; Ic3k 2019 Proceedings of the 11th International Joint Conference on Knowledge Discovery Knowledge Engineering and Knowledge Management; Icete 2019 Proceedings of the 16th International Joint Conference on E Business and Telecommunications; Interactive Learning Environments; International Conference on Information Networking; International Journal of Advanced Science and Technology; International Journal of Information and Education Technology; International Journal of Network Management; Journal Of Advanced Research in Dynamical and Control Systems; Knowledge Engineering Review; Open Review Of Educational Research; Procedia Computer Science; Proceedings of the 2019 International Conference on Cyber Enabled Distributed Computing and Knowledge Discovery Cyberc 2019; Proceedings of the 9th International Conference on Information Technology in Medicine and Education Itme 2018; Proceedings of the 12th International Conference on Global Security Safety and Sustainability Icgs3 2019; Proceedings of the International Conference on Intelligent Engineering and Management Iciem 2020; Proceedings of the 15th International Conference on Cognition And Exploratory Learning in the Digital Age Celda 2018; Research and Practice in Technology Enhanced Learning.

We can say that there was a sharp interest in research on the blockchain system in Higher Education.

In Table 2 we analyze for the Scimago Journal & Country Rank (SJR), the best quartile and the H index by publication. Interactive Learning Environments with 1,230 (SJR), Q1 and H index 38. There is a total of five journals on Q1, four journals on Q2 and. Journals from best quartile Q1 represent 16% of the 31 journals titles.

As shown in Table 1, the significant majority of articles on the blockchain system in Higher Education still lack data.

The subject areas covered by the 31 scientific articles were: Computer Science (31); Engineering (16); Social Sciences (10); Mathematics (6); Business, Management and Accounting (3); Decision Sciences (3); Materials Science (3); Energy (2); (Arts and Humanities (1); Chemical Engineering (1); Medicine (1); Physics and Astronomy (1); and Psychology (1). 

The most quoted article was “EduCTX: A blockchain-based higher education credit platform” from EduCTX: A blockchain-based higher education credit platform with 116 quotes published in the IEEE Access with 0,780 (SJR), the best quartile (Q1) and with H index (86). The published article presents a prototype for implementing the environment, based on the open-source platform Ark Blockchain.

**Figure 2:**
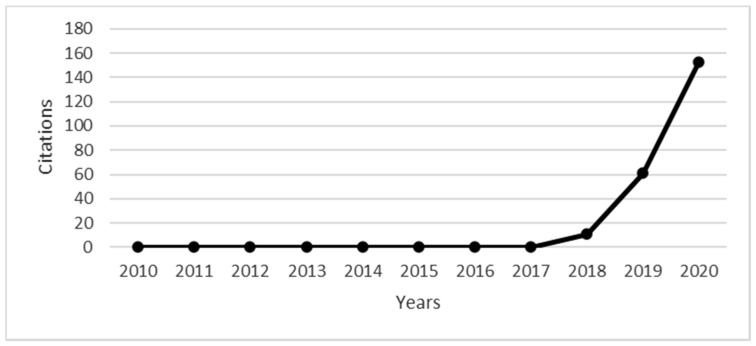
Evolution of citations between 2010 and 2020. (Source: own elaboration)

In Figure 2 we can analyze the evolution of citations of articles published between 2010 and 2020. The number of citations shows a net positive growth with an R2 of 43.5% for the 2010–2020 period, with 2020 peaking at 153 citations.

The h-index was used to ascertain the productivity and impact of the published work, based on the largest number of articles included that had at least the same number of citations. Of the documents considered for the h-index, six have been cited at least six times.

In Appendix A, Table A1, citations of all scientific articles until 2020 are analyzed; 12 documents were not cited until 2020. 

Appendix B, Table A2, examines the self-quotation of documents until 2020, in which was identified self-quotation for a total of eight self-quotations.

**Figure 3:**
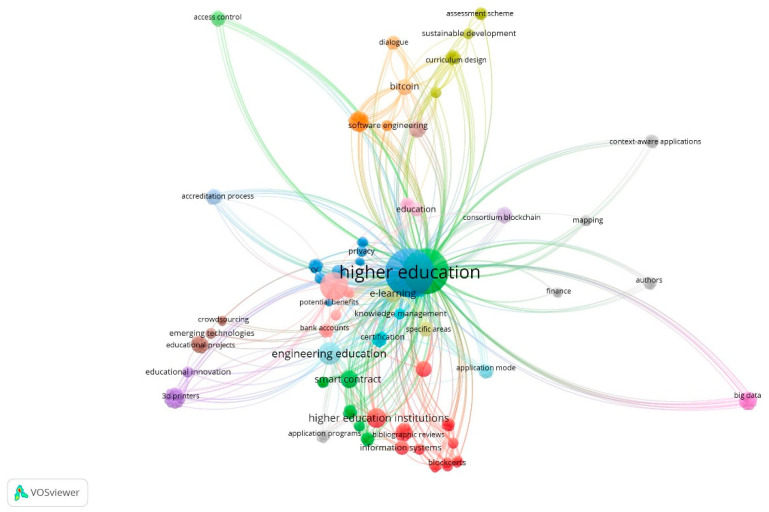
Network of all keywords. Source: own elaboration.

In Figure 3, a bibliometric analysis was carried out to analyse and identify indicators on the dynamics and evolution of scientific information using the main keywords. The analysis of the bibliometric research results using the scientific software VOSviewe aims to identify the main keywords of research in sustainability as a marketing strategy.

**Figure 4:**
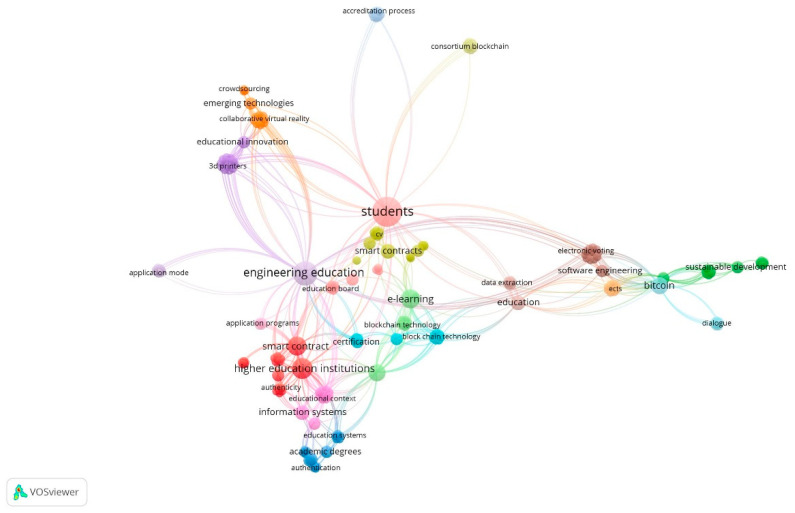
Network of Linked Keywords. Source: own elaboration.

**Figure 5:**
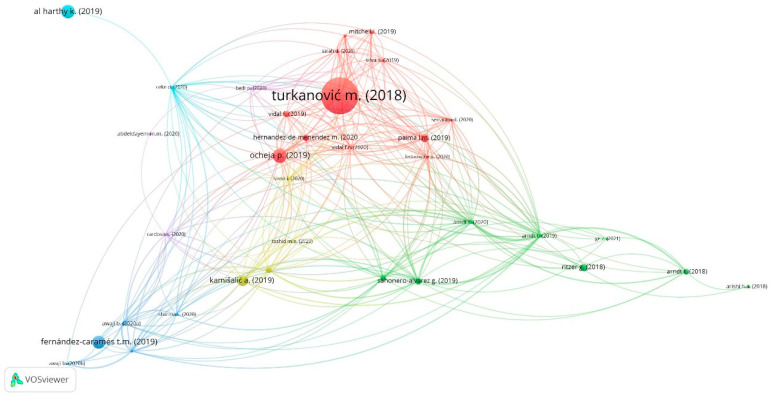
Networks bibliographic coupling. Source: own elaboration.

The linked keywords can be analysed in Figure 4, making it possible to clarify the network of keywords that appear together/linked in each scientific article, allowing to know the topics analysed by the research and to identify future research trends. In Figure 5, a profusion of networks bibliographic coupling of publications’ researchers is presented.

## 5. Discussion

### 5.1. Research Themes

An analysis was carried out to analyze the reviewed studies and define thematic areas of research that embody key issues dealt by the above-mentioned reviewed studies (Figure 3 and Figure 4). These main thematic areas comprehend higher education and students, which encircled noteworthy subthemes. The review suggests that research efforts have been aimed at building new knowledge to improve sustainable innovation and smart contracts both for students and HEIs, based upon the effectiveness and efficiency in terms of processes and data management of Blockchain Technology. In this way, the main themes that come out in this LRSB and where some authors are key [18] (Figure 5) are the following.

#### 5.1.1. Higher Education Institutions

The results of the review point out that research in the area of blockchain in higher education has been largely aimed at the developing of concepts related with the application of Blockchain in organizing HEIs. This technology has evolved in these institutions around its informative systems, bibliographic reviews, and knowledge organization in general through smart contracts. Indeed, the topic is posed on the digitization of degree certificates and academic credits for higher education in developing countries such as Brazil, in order to ease the organization of their education system, which, in conjunction with smart contracts, enables the reliable and decentralized issuance of degree certificates [21]. Attention has been paid to the issue of avoiding counterfeit or falsified certificates, by underscoring blockchain technology and smart contracts, to implement a decentralized verification solution for trustworthy qualifications (diplomas or certificates). It allows HEIs to register the certificates they issue in the blockchain, while recruiting organizations to check the authenticity and integrity of these certificates [22]. Therefore, extant studies have incorporated blockchain in higher education, highlighting the split of data into secured blocks, ensuring privacy in secure data transactions [30] and improving, therefore, HEIs e-governance [3].

Hence, extant research has focused on Blockchain as a new platform for keeping track of learning achievements beyond transcripts and certificates, specifically in how learning or teachings were conducted and attained by maintaining digital hashes of learning activities through smart contracts. This technology, based on a platform of learning logs, enables learners to move their learning records from one institution to another in a secure way. Furthermore, it enables existing learning data analytic platforms to access the learning logs from other institutions with the permission of the learners and/or institution, who originally owned the logs [31]. For example, one can build either a blockchain-based platform to create and store contracts in between students and their higher education sponsors, creating and storing transaction in distributed secure ledgers [32], or an editorial platform to eliminate the problems of copyright management, piracy, and lack of transparency between publisher and author [33]. In the end, existing literature has made significant developments in proposing guidelines including blockchain integration levels in higher education that, combined with data extraction, can expand the area of education while ameliorating credibility and independence of HEIs [34].

Moreover, the suggested decentralization technology allows for a secure exchange of academic marks between HEIs, for instance in the case of student mobility programs (i.e., the EU Erasmus Program) [34]. As a result, a verifiable record of achievements also enables students to further present academic accomplishments to employers, within a trusted framework, thus facilitating communication between industry and students for employment purposes and simplify the search for appropriate potential employees for the job [33].

#### 5.1.2. Smart Contracts

The issue of smart contracts has been central for the research on Blockchain, both to HEIs and to students. Blockchain has been enhanced as a tool of smart contracts in its immutability, provenance, and peer execution that can provide new levels of security, trust, and transparency to e-learning. Blockchain technology is introduced as a based e-learning platform developed to increase transparency in assessments and facilitate curriculum personalization in higher education context. It enables the automation of assessments and issues credentials, benefiting thus both students and teaching staff [4]. In fact, in its distributed ledger, Blockchain can remove the need for a trusted third party to facilitate transaction and can improve smart contracts by Blockchain-based protocols, which can automatically enforce a contract in students throughout multiple levels of administration. It therefore opens higher education to a wider range of learners [17], while setting routines for the way professors deliver contents, manage courses and assess student work, in knowledge management [19].

Such Blockchain-based protocols can be achieved by sharing and issuing digital certificates through a chain of records using a public key infrastructure for identity management [21]. For example, it can be achieved by implementing a decentralized verification solution for higher education certificates involving: the consortium smart contract, the HEIsmart contract, the HEIclient, the recruiter app, and the consortium app [22]. It can be accomplished as a new platform for keeping track of learning achievements through digital hashes, enabling learners to securely move their learning records from one institution to another [31]. Blockchain can therefore be used in addressing issues for students and remaining stakeholders with regard to accumulating shareable resources through a transparent process for everyone [35].

#### 5.1.3. Privacy and Accreditation Process

Privacy and accreditation process constitute recurrent themes for research on Blockchain in HEIs and with respect to the authentication of academic degrees for students, as it discusses the problem of corrective operations done without altering the existing data and guaranteeing privacy by building new solutions through distributed ledgers [36,37]. It intends to use Blockchain and its characteristics of immutability to ensure the issue of academic certificates, proposing a mechanism to revoke digital diplomas that may have been issued incorrectly [16]. Existent research highlights the studies which cover the possibility of adopting Blockchain in education institutions for dividing the data into secured blocks, thus ensuring privacy in data transactions of student certifications [30], in which the focus is on ensuring the authenticity of degrees, while keeping accurate records [6], the control of student credits for completed courses towards a more transparent system [18] the building of a trusted achievement record for employers [20], dividing the data into secured blocks, while guaranteeing privacy in data transactions and student certifications [30]. A decentralized model of confidence for transaction of content, teaching and competences, consensually shared is advocated [37].

#### 5.1.4. Knowledge Management, Certification and Engineering Education

Some scholars have focused on the incorporation of a Blockchain platform into engineering education. These works review Blockchain technologies that are transforming engineering education. It presents descriptions, examples of currently available tools, case studies, benefits, challenges, time to adoption, results, future development, and suggestions for the implementation of Blockchain in Engineering education. The focus is aimed at using this technology for assisting students in their acquisition of technical knowledge and development of competencies in Engineering and Science education, in which the advantages are immense in an education more based on experimentation [9]. Such research is directed either towards dividing the data into secured blocks for data transactions [30], or in the way knowledge is managed, produced, and shared in terms of delivering contents and managing courses [19].

#### 5.1.5. Innovation in Education

Most of the research in this issue has focused on the innovation of developed technology through technical improvements. A Blockchain platform, for example, is proposed to ease access to high-quality education materials, a decentralized marketplace for offering, acquiring, discussing, and improving education resources across different universities. It innovatively allows control via on-chain license terms in tracing the evolution of encrypted containers, while accumulating bundles of shareable resources and user records to improve the quality of content [35]. This trend of research emphasizes diverse systems amongst academic projects, therefore improving higher education in areas such as collaborative virtual reality, 360° courses, Blockchain for digital credentials, and digital tutors, in the process of experiencing and creating new solutions [36]. It is noteworthy to mention the projects involving Blockchain technologies that are transforming engineering and science education, towards more experimentation [9] and information and Internet technology [2,12,36].

#### 5.1.6. Emerging Technologies and Educational Projects

Scholarly attention has pointed out emerging technologies such as potential opportunities related with utilization in human resources management and adoption challenges that may hamper it [3]; a Blockchain-based platform to create and store contracts in between students and their higher education sponsors, in which the fund will be managed by fundraisers, who will hold the distributed ledgers and act as the miners in the Blockchain network, creating and storing transaction in distributed ledgers [37]; editorial platforms to remove the constraints that publishers, writers, translators and readers currently deal with, such as the lack of transparency of the actual number of sales, augmenting therefore the opportunity to publish educational content and be fairly rewarded [38]; and, last but not least, the creation of tutorials for Bitcoin that allows for learners from technical and legal backgrounds to be taught through Blockchain in a digital forensics programme and by a higher education provider [39]. In sum, Blockchain provides a user-friendly environment for all stakeholders [40], while guaranteeing privacy [41] through a decentralized model of confidence [42].

#### 5.1.7. E-Learning

Some studies in the extant literature have focused on understanding how Blockchain can augment the efficiency of e-learning processes. As e-learning environments are expanding from only virtual learning environments to both virtual and physical ones, the expertise and virtual learning education, namely in e-learning education increases as well, introducing therefore the structures of Blockchain knowledge apps for virtual learning education [1]. Blockchain technology in its smart contract features of immutability and peer execution allows the provision of new levels of trust, security, and transparency to e-learning, therefore increasing transparency in assessments and easing curriculum building in higher education. Moreover, it enables the automation assessments and issue credentials and, while pedagogically neutral and content-neutral, it benefits both students and teaching staff, thus increasing trust in online higher education procedures [4].

#### 5.1.8. Document Organization

Prior research has, to some extent, focused on the need to ensure legally compliant processing, sharing, and handling of higher education documentation. Blockchain technology allows the creation distributed applications involving multiple organizations in which transactions and data are not under the control of a central authority, but stored in a distributed public ledger, in an immutable format in such a way that they can be verified by participants [11]. Such features ensure that, for example, the public verification of students’ answers in higher education and answer records cannot be corrupted, while it can either be traced and protected by group signature [14] or stored, drawing on intellectual effort and ensuing reputational reward [43]. In addition, these characteristics are particularly useful in developing countries, in which the adoption of new technologies in this field is scarce and the use of Blockchain is important in terms of connection between student and faculties to, innovatively, decentralize and validate records [15].

### 5.2. Findings: The Research Building for Blockchain Technology in the Higher Education Sector to Date 

The present study carried out a LRSB on Blockchain in higher education in order to understand its current position nowadays. To attain this goal, the up to date research on Blockchain applications in higher education was addressed by outlining the major contribution trends in the issue. In this way, we discuss the findings based on the research questions and the results obtained. What is the research building for Blockchain technology in the higher education sector to date?

We can notice that Blockchain application in higher education is over enhanced in literature in terms of two major themes: its applications for HEIs and for students. It is significant to mention that Blockchain in higher education has been addressed from different but intertwined viewpoints and an agreement on its classification has not yet been achieved. 

Firstly, literature posits that Blockchain provides a digital and decentralised learning infrastructure to all stakeholders through learning platforms [25], of relevant data security for administrative use [33] and with flexible design in terms of shared compliance for decision making [9]. It allows us, therefore, to build links between diverse universities in academic programmes [34], to improve governance by supporting the management in higher education with innovative resource allocation [3] and to augment its human resources effectiveness and digital competency [3]. Blockchain literature therefore favours technical knowledge and innovation in higher education. A better coordination between universities in sharing educational resources [35], experimentation [9] and new creative solutions [2,12,32], for example, is suggested. It is a new technology able to bring up improvements in what comes to managing information, while ensuring its privacy and authenticity for all stakeholders, in particular HEIs and students, which are considered central actors in the at once organizational and technological process. 

Secondly, with regard to HEIs, Blockchain allows improving technology in terms of securing and sharing authentic digital certificates [16], whereas for students, it guarantees the safe sharing of critical academic data between them and key agents such as sponsors, editors, loan providers and employers [20]. Additionally, Blockchain application is particularly useful for students, with regard to the accreditation process and knowledge management in engineering education, related with the acquisition of technical knowledge through experimental methodologies [9,19]. Besides HEIs and students, most actors interested in this technology also include teachers, researchers, government, and the industries of digital platforms and education itself [19].

Thirdly, literature suggests Blockchain technology as a huge contribution for all stakeholders as it enables the facilitation of knowledge organization, for example in the case of issuing certificates [20,21,30], to prevent counterfeit or falsified documents [22] and ensuring its privacy [3]. Hence, it allows, safely and by a decentralized platform, moving of students’ learning records from one institution to another [18,31], between sponsors and students [37,40], employers and students [20,33]. Furthermore, it facilitates student mobility programs [34] and share pieces of writing between authors and editors [34]. The applications are numerous and allow partnerships and collaborative customer relationships that, in the end, enhance the whole educational ecosystem. 

Fourthly, literature suggests that the teaching interest on Blockchain relies on the e-learning and digital platforms and contents for teaching, therefore improving the didactics reliant on data from shared sources [4] and innovation through educational projects [12], thus promoting academic curriculum [4], organizational innovation [35] and experimental learning [9]. In the end, innovative teaching allows contributing to the added value of stakeholders overall. Blockchain technologies are quite innovative as they create and store transactions within distributed ledgers and between different educational agents [3,37,38,39], thus ameliorating knowledge applications for e-learning [1] and trust in online higher education procedures [4]. 

Fifthly, this technology is presented as central to intermediate contexts, of scarce resources, wherein the process of organizing documentation and support students is in its early stages and needs to keep up with these innovative technologies [15,40]. In this way, according to the literature, Blockchain applications in higher education rely on varying environments of distinct actors, processes/structures and cannot be analysed detached from it. Instead, it rather needs to be integrated into distinct organizational protocols and learning practices [4]. Moreover, these protocols, attained by a chain of decentralized verified records [21,22], are based upon secured platforms of digital hashes [31,40] and allow corrective operations without altering the existing data [6,16,30]. Likewise, it allows to process and manage documentation innovatively, involving varying organizations in which contents are distributed in such a way that they are kept unchanged and can be verified [11], traced [14] and validated [15]. 

Sixthly, Blockchain technology comes up mostly through the form of diverse smart contracts that favour learning, data sharing and organization of documentation [11], whilst ensuring privacy to the accreditation process. These varying contracts of distinct interests and from different contexts, by means of automatically discarding the need for an intermediary, ensure trust and transparency to e-learning protocols for all those involved [4,18,19], while augmenting the number of potential learners [17]. 

Finally, one can conclude that the literature presents Blockchain technology through varying perspectives, although it posits a consensus regarding its advantages when applied to higher education. In sum, it is a technology achievable and timely in higher education because on the one hand it matches the researchers/students’ interests, who expect to seize the opportunity to study, irrespective the hurdles of time and space, while acquiring digital skills for the labour market [12,20]; and, on the other hand, it addresses HEIs’ concerns in developing content delivering, enhancing educational quality and replacing the traditional educational services [15]. The introduction of Blockchain infrastructures is therefore opportune, in terms of learning content, innovative lecturing and research, although it means a significant change, involving multiple limitations and challenges that are discussed bellow. 

### 5.3. Findings: The Current Challenges and Future Research Paths in the Theme

The results of this study highlight the challenges identified by previous research on Blockchain, which point out mainly its technical limits [10]. The reviewed articles have principally focused on the need to develop new algorithms [14] and frameworks [33] for implementing Blockchain in higher education. Building upon the review, we have listed the existing limitations in seven key issues, which comprehend usability, scalability, platform and algorithm suitability, societal constraints, cost, privacy, and immutability. 

Firstly, Blockchain technology usability is a main limitation in higher education. The technology jargon is relatively new and lacks development. It is noteworthy that Blockchain includes very different specifications that can make it difficult for end users [40]. Furthermore, users should deal with diverse issues that complicate security, such as primary keys and public keys [21]. Hence, Blockchain usability should be improved through new design interfaces that are more responsive to users, while training in its use should be delivered to professors, students, and staff [36], as the majority of the academic community is unaware of this technology [40]. Therefore, further studies on Blockchain usability are required. 

Secondly, scalability regards the way the rising number of users and transactions can affect access to Blockchain network. Studies have posited that high usage ratios may influence the intrinsic stability of a framework and ensuing performance [32]. As a campus can cover a large number of users requesting smart services, it is essential for the application to be easily scalable, to proportionally adapt its performance [5]. Prior system architecture should consider this issue with regard to education, as it is difficult to predict the path of Blockchain technology in terms of potential future scale [32]. It needs to add functionality to the systems while scaling them up with a larger quantity of data [41], to explore, by smart contracts, its capacity with respect to higher education learning outcomes [4] and to ensure that the platform can be implemented as a wide-reaching system [31,35]. It is therefore an issue that requires further research to understand how many participants, assets and increasing in transactions would impact the access latency [2]. 

Thirdly, the application of different shared algorithms may also be considered constraints for Blockchain deployment [32], while there is a lack of smart campus standards with respect to a common framework, capable of enabling an effective Blockchain-based higher education ecosystem [5]. The small integration of smart campus applications makes it necessary to design devices that allow for switching between new architectures such as big data, machine learning, deep learning, and new communications platforms [5,16]. Interchangeable platforms and algorithms allow for comparing curricula from different courses/degrees so that students can move from one higher education institution to another and resume their studies in an equivalent degree [21]. Future research therefore needs to develop an integrated model and ensuing value chain, from the moment students enter higher education to the moment they get their diplomas [6]. It should focus on making the application more efficient in connecting platforms [13], ensuring decentralization [22] and a potential ecosystem among distinct software [35] of universal standard file formats [16]. It may potentially evolve into a united, simplified and globally ubiquitous higher education credit and grading system through novel Blockchain platforms able to augment its ensuing scalability and usability [18]. One will have to keep compatibility issues in mind to ensure service flexibility by multiple services [5], while safeguarding transaction data of sensitive academic information [14]. 

Fourthly, our review suggests that societal constraints such as the lack of enthusiasm around Blockchain with regard to the ethical and secure use of data may constitute a substantial challenge that may hinder the adoption of Blockchain by HEIs. On the one hand, it is difficult to persuade education actors to implement Blockchain systems because of its novelty, which could be mitigated through appropriate training and the development of usable Blockchain-applications [15]. Moreover, Blockchain reduces university administrative staff-related expenses and therefore, university administrations may resist its implementation [7]. On the other hand, the extensive adoption of Blockchain systems requires political support amidst a context of indistinct legal status [16] when it comes to deciding, for example, the Blockchain versions for governance decentralization [22]. In so doing, both the will to adopt the platform and the legal mechanisms to support it play important roles in terms of its successful deployment. For example, some scholars posit that the wide use of Blockchain for IP commercialization purposes and the increasing number of conflicts related to the inclusion of IP objects in various registries require prompt progress on mitigating its legal risks [38]. In this way, further studies in terms of legal frameworks on Blockchain transactions are needed. 

Fifthly, another limitation concerns the costs involved in Blockchain transactions, because dealing with large amounts of academic data on the platform may increase costs. It principally relates to the time, resources, and monetary costs associated with carrying out a Blockchain framework, in which it is necessary to carefully monitor costs of usage in traditional education systems [12,20]. This can inhibit the adhesion of universities, because, for example, one cannot predict the rate of transactions in Bitcoin in the long term [6]. Current solutions, such as legacy credential verification systems, are awkward and are neither time- nor cost-efficient, whereas educational institutions can reduce costs by sharing infrastructure, academic programmes and services, for instance, in online education using Blockchain technology [15]. Research is thus needed in the delivering of a more user-friendly and efficient platform that effectively integrates into the existent credential verification ecosystem [33].

Sixthly, it is important to consider how data can be securely accessed and used while maintaining privacy [32]. Blockchain systems use both private and public keys to protect user identities, but since public keys are visible they cannot ensure transactional privacy [21]. Additionally, students may lose all academic diplomas whenever they lose the secret key information required to prove ownership [16]. Thus, Blockchain privacy mechanisms present weaknesses that may lead to abuse [14], being therefore central to protect the identities of users by mapping a linkage between the pseudonyms and real identities [11]. Further research should add greater emphasis on accurate mechanism of learning logs and ensuing privacy measures are needed to build standardized formats for permissions on the Blockchain [31], such as a distributed storage medium to avoid forged diplomas [14].

Finally, Blockchain’s immutability consists of its impossibility for the data stored in the blocks to be changed, which is a critical feature of Blockchain technology [4,16]. Nonetheless, immutability is a key limitation to using Blockchain technology for education, for instance in the case of diploma revocation, in which the diplomas that are stored on the Blockchain cannot be changed [16]. For this reason, immutability can reduce Blockchain use with respect to students’ sensitive data as in the case of admissions, certificate/degree verification and exams/assessments that require the right to delete. The scalability and performance efficiency of a framework is, in this way, affected by requirements for continual upgrades by the utilized system [2]. Further research is therefore needed with regard to the trade-off between resource protection and flexibility [35]. 

## 6. Conclusions

This study aimed to understand the extent of Blockchain application in the higher education field. To address this goal, an LRSB was carried out on a respected digital database following distinct protocols to point out suitable pieces of writing to be reviewed. The findings were used to sum up existing knowledge on Blockchain application in the distinct domain of higher education and summarize current thematic trends of academic work in this issue. The principal and emergent insights and challenges comprehend compatible digital platforms to safely share and organize data, flexible smart contracts, affordable innovative projects, and privacy/learning issues to all the stakeholders involved in the administrative and learning processes in higher education.

These insights should be considered in light of their limitations. First, the review focused exclusively on articles appearing in peer-reviewed publications available in the SCOPUS database of scientific articles published by 2020. Second, the review considered Blockchain as an umbrella keyword and did not consider compatible keywords, such as smart contracts. These limitations may be addressed in future research by considering supplementary interchangeable keywords, to expand the scope of information. 

This study concludes that it is required to find solutions for performance and security-related issues, such as interoperability between distinct platforms/algorithms and secure access-control in the light of the potential adoption of smart contracts in higher education. Furthermore, researchers should adopt a holistic perspective of Blockchain deployment in order to build legally and culturally compliant ecosystems, because culture is central when it comes to develop customized and collaborative higher education solutions.

To sum up, our contribution is a detailed review of the recent literature on the application of Blockchain in higher education, together with its main challenges. These insights will assist researchers in understanding the main areas of research and identify further research paths.

## Figures and Tables

**Table 1:** Screening Methodology.

Database Scopus	Screening	Publications
Meta-search	keyword: Blockchain	17,975
Inclusion Criteria	keyword: Blockchain and education	545
Screening	keyword: Blockchain and education Exact Keyword: Higher Education Published until December 2020	37

Source: own elaboration.

**Table 2:** Scimago journal & country rank impact factor.

Title	SJR	Best Quartile	H Index
Interactive Learning Environments	1.230	Q1	38
IEEE Access	0.780	Q1	86
Research And Practice In Technology Enhanced Learning	0.690	Q1	11
Applied Sciences Switzerland	0.420	Q1	35
Open Review Of Educational Research	0.310	Q1	8
International Journal On Interactive Design And Manufacturing	0.460	Q2	20
Knowledge Engineering Review	0.450	Q2	60
International Journal Of Network Management	0.380	Q2	27
European Journal Of Contemporary Education	0.380	Q2	9
Communications In Computer And Information Science	0.190	Q3	45
Advances In Intelligent Systems And Computing	0.180	Q3	34
Journal Of Advanced Research In Dynamical And Control Systems	0.130	Q3	17
International Journal Of Advanced Science And Technology	0.110	Q4	3
Procedia Computer Science	0.340	- *	59
International Conference On Information Networking	0.280	- *	24
ACM International Conference Proceeding Series	0.200	- *	109
Iberian Conference On Information Systems And Technologies Cisti	0.180	- *	13
International Journal Of Information And Education Technology	- *	- *	1
2018 World Engineering Education Forum Global Engineering Deans Council Weef Gedc 2018	- *	- *	- *
2019 42nd International Convention On Information And Communication Technology Electronics And Microelectronics Mipro 2019 Proceedings	- *	- *	- *
2019 4th Mec International Conference On Big Data And Smart City Icbdsc 2019	- *	- *	- *
2020 2nd Conference On Blockchain Research And Applications For Innovative Networks And Services Brains 2020	- *	- *	- *
2020 43rd International Convention On Information Communication And Electronic Technology Mipro 2020 Proceedings	- *	- *	- *
Csedu 2020 Proceedings Of The 12th International Conference On Computer Supported Education	- *	- *	- *
Ic3k 2019 Proceedings Of The 11th International Joint Conference On Knowledge Discovery Knowledge Engineering And Knowledge Management	- *	- *	- *
Icete 2019 Proceedings Of The 16th International Joint Conference On E Business And Telecommunications	- *	- *	- *
Proceedings 2019 International Conference On Cyber Enabled Distributed Computing And Knowledge Discovery Cyberc 2019	- *	- *	- *
Proceedings 9th International Conference On Information Technology In Medicine And Education Itme 2018	- *	- *	- *
Proceedings Of 12th International Conference On Global Security Safety And Sustainability Icgs3 2019	- *	- *	- *
Proceedings Of International Conference On Intelligent Engineering And Management Iciem 2020	- *	- *	- *
Proceedings Of The 15th International Conference On Cognition And Exploratory Learning In The Digital Age Celda 2018	- *	- *	- *

Note: * data not available. Source: own elaboration.

## Data Availability

Not applicable.

## Appendix A

**Table A1:** Overview of document citations period 2010 to 2020.

Documents		<2010	2011	2012	2013	2014	<2015	2016	2017	2018	2019	2020	Total
Mostla for engineering education: Part 1…	2020	-	-	-	-	-	-	-	-	-	-	2	2
Blockchain-based applications in higher education: A systema…	2020	-	-	-	-	-	-	-	-	-	-	1	1
Revocation Mechanisms for Academic Certi_cates Stored on a…	2020	-	-	-	-	-	-	-	-	-	-	1	1
Technologies for the future of learning: state of the art	2020	-	-	-	-	-	-	-	-	-	-	4	4
Blockchain-based transcripts for mobile higher-education…	2020	-	-	-	-	-	-	-	-	-	-	-	1
Trends ofglobal _ntech education practices and the gcc pers…	2020	-	-	-	-	-	-	-	-	-	-	1	1
A Secure and Practical Decentralized Ecosystem for Shareable…	2020	-	-	-	-	-	-	-	-	-	-	1	1
A blockchain-enabled e-learning platform	2020	-	-	-	-	-	-	-	-	-	-	3	3
Managing lifelong learning records through blockchain	2019	-	-	-	-	-	-	-	-	-	4	13	17
Towards next generation teaching, learning, and context-awar…	2019	-	-	-	-	-	-	-	-	-	-	15	15
Analysis of blockchain technology for higher education	2019	-	-	-	-	-	-	-	-	-	-	3	3
Creating student’s pro_le using blockchain technology	2019	-	-	-	-	-	-	-	-	-	1	2	3
DAppER: Decentralised Application for Examination Review	2019	-	-	-	-	-	-	-	-	-	1	1	2
The upcoming Blockchain adoption in Higher-education: Requir…	2019	-	-	-	-	-	-	-	-	-	1	15	16
Blockchain and peace engineering and its relationship to eng…	2019	-	-	-	-	-	-	-	-	-	2	2	4
An overview of blockchain for higher education	2019	-	-	-	-	-	-	-	-	-	-	1	1
Editorial platform in blockchain for application in higher e…	2019	-	-	-	-	-	-	-	-	-	-	1	1
A Preliminary Review of Blockchain-Based Solutions in Higher…	2019	-	-	-	-	-	-	-	-		1	9	10
Blockchain and smart contracts for higher education registry…	2019	-	-	-	-	-	-	-	-	-	-	-	6
Research on Online Quiz Scheme Based on…	2018	-	-	-	-	-	-	-	-	-	3	4	7
EduCTX: A blockchain-based higher education credit platform	2018	-	-	-	-	-	-	-	-	11	4	60	116
The velvet cage of educational con(pro)sumption	2018	-	-	-	-	-	-	-	-	-	-	5	5
Block chain technology and its applications for virtual educ…	2018	-	-	-	-	-	-	-	-	-	-	1	1
Empowering university students with blockchain-based transcr…	2018	-	-	-	-	-	-	-	-		1	2	3
Bitcoin forensics: A tutorial	2016	-	-	-	-	-	-	-	-	-	1		1
	Total	-	-	-	-	-	-	-	-	11	61	153	225

## Appendix B

**Table A2:** Overview of document self-citation period 2010 to 2020.

Documents		<2010	2011	2012	2013	2014	<2015	2016	2017	2018	2019	2020	Total
Managing lifelong learning records through blockchain	2019	_	_	_	_	_	_	_	_	_	_	1	1
Analysis of blockchain technology for higher education	2019	_	_	_	_	_	_	_	_	_	_	1	1
Research on Online Quiz Scheme Based on Double-Layer Consort…	2018	_	_	_	_	_	_	_	_	_	1	2	3
EduCTX: A blockchain-based higher education credit platform	2018	_	_	_	_	_	_	_	_	_	_	2	2
Block chain technology and its applications for virtual educ…	2018	_	_	_	_	_	_	_	_	_	_	1	1
		_	_	_	_	_	_	_	_	_	1	7	8
